# Recovery and Recurrence in Bell’s Palsy: A Propensity Score-Matched Comparative Study Across ENT, Pain Medicine, and Traditional Korean Medicine

**DOI:** 10.3390/medicina61071239

**Published:** 2025-07-09

**Authors:** Jaeyoon Chung, Eunsung Park, Jin Lee, Cheol Lee

**Affiliations:** 1 Department of Anesthesiology, Wonkwang University Sanbon Hospital, Gunpo 15865, Republic of Korea; chungjaeyoon010@gmail.com; 2 Department of Neurosurgery, School of Medicine Hospital, Wonkwang University, 895 Muwang-ro, Iksan-si 54538, Republic of Korea; silverstar0401@gmail.com; 3 Department of Otorhinolaryngology-Head and Neck Surgery, School of Medicine Hospital, Wonkwang University, Iksan-si 54538, Republic of Korea; 4 Department of Anesthesiology and Pain Medicine, School of Medicine Hospital, Wonkwang University, 895 Muwang-ro, Iksan-si 54538, Republic of Korea

**Keywords:** Bell’s palsy, treatment outcome, recurrence risk factors

## Abstract

*Background and Objectives*: Bell’s palsy, characterized by acute idiopathic facial nerve paralysis, exhibits variable recovery outcomes influenced by treatment timing, modality, and patient comorbidities. This study aimed to compare the effectiveness of corticosteroid-based treatment (Ear, Nose, and Throat [ENT]), nerve blocks/physical therapy (Pain Medicine), and acupuncture/herbal medicine (Traditional Korean Medicine [KM]) and identify predictors of recovery and recurrence. This retrospective cohort study leverages South Korea’s pluralistic healthcare system, where patients choose specialties, to provide novel insights into departmental treatment outcomes. *Materials and Methods*: We analyzed 600 patients treated within 72 h of Bell’s palsy onset (2010–2024) at Wonkwang University Hospital, South Korea, using propensity score matching (PSM) (1:1:1) for age, sex, comorbidities, and initial House–Brackmann (HB) grade. The primary outcome was complete recovery (HB grade I) at 6 months; secondary outcomes included recovery time, recurrence, complications, and patient satisfaction. Multivariate logistic regression identified predictors. *Results*: The ENT group achieved the highest complete recovery rate (87.5%, phi = 0.18) versus Pain Medicine (74.0%) and KM (69.5%) (*p* < 0.001), with the shortest recovery time (4 weeks, Cohen’s d = 0.65 vs. KM). Synkinesis was lowest in the ENT group (6.0%). ENT treatment (OR: 1.75; 95% CI: 1.29–2.37) and early corticosteroid application (OR: 1.95; 95% CI: 1.42–2.68) predicted recovery. Hypertension (OR: 4.40), hyperlipidemia (OR: 8.20), and diabetes (OR: 1.40) increased recurrence risk. Subgroup analyses showed that ENT treatment was most effective for severe cases (HB grade IV: 90% recovery vs. 65% in KM, *p* < 0.01). *Conclusions*: Corticosteroid-based treatment (ENT) yielded superior recovery outcomes. Comorbidity management is critical for recurrence prevention. Early ENT referral and integrated care models are recommended to optimize outcomes in diverse healthcare settings.

## 1. Introduction

Bell’s palsy is an acute, idiopathic peripheral facial nerve paralysis that typically presents as sudden unilateral facial weakness [1,2,3]. While most patients recover spontaneously, 15–30% experience incomplete recovery, leading to facial asymmetry, synkinesis (involuntary muscle movements during voluntary actions), and psychological distress, which significantly impact quality of life [1,3,4]. It is a common cranial nerve disorder, with an annual incidence of 15–30 per 100,000 globally and up to 40 per 100,000 in South Korea [1,5]. Proposed etiologies include viral reactivation (e.g., herpes simplex virus type 1), ischemia, inflammation, and autoimmune mechanisms [1,2,6]. Although spontaneous recovery occurs in 70–85% of cases, timely intervention is critical to minimize long-term sequelae, particularly in severe cases or patients with comorbidities [3,4].

Current treatments primarily involve corticosteroids and antivirals in the acute phase, yet alternative modalities like acupuncture and nerve blocks are increasingly being utilized, particularly in integrative healthcare systems [7,8,9,10,11]. However, the optimal treatment strategy remains uncertain due to limited comparative studies across specialties. Few studies have directly compared outcomes among Ear, Nose, and Throat (ENT), Pain Medicine, and Traditional Korean Medicine (KM) departments, despite their distinct approaches. Moreover, the role of comorbidities such as hypertension, diabetes, and hyperlipidemia in recurrence is underexplored in multivariate models, and patient satisfaction metrics are rarely evaluated across specialties [12,13]. This study addresses these gaps by evaluating recovery, recurrence, and satisfaction in a propensity-score-matched cohort.

We hypothesized that corticosteroid-based treatment in the ENT department would yield faster and more complete recovery with fewer complications compared to Pain Medicine or KM. We also aimed to identify clinical predictors of recovery and recurrence, focusing on modifiable risk factors to inform integrated care strategies in settings where multiple therapeutic disciplines coexist, such as South Korea’s pluralistic healthcare system.

### Korean Healthcare Context

South Korea’s healthcare system allows patients to directly choose specialties, including ENT for pharmacological treatment, Pain Medicine for nerve blocks or physical therapy, and KM for acupuncture and herbal medicine [11,14]. This unique structure facilitates comparative studies of treatment modalities, as patients self-select departments based on their preferences or cultural beliefs. Prior studies, such as that by Jeong et al. (2022), have highlighted specialty-specific care patterns for facial palsy in Korea, but robust comparative outcome data are lacking [11]. This study leverages real-world data to address this gap, providing insights into departmental approaches and their implications for global Bell’s palsy management [11,14].

## 2. Materials and Methods

### 2.1. Study Design and Setting

This retrospective cohort study was conducted at Wonkwang University School of Medicine Hospital, a tertiary academic hospital in South Korea, using electronic medical records from 1 January 2010 to 31 December 2024. The study was approved by the Institutional Review Board (WKUH-2025-04-002-21), and informed consent was waived due to the retrospective nature of the study.

#### Diagnostic Criteria

Bell’s palsy was diagnosed based on sudden unilateral facial weakness or paralysis without identifiable causes, confirmed by clinical evaluation by otolaryngologists or neurologists. Where necessary, imaging (e.g., MRI) or electrophysiological testing was used to exclude other etiologies, such as Ramsay Hunt syndrome, stroke, or tumors. This standardized approach ensured diagnostic consistency across treatment groups [2].

### 2.2. Study Population

Eligible patients were adults (aged 18 years or older) diagnosed with Bell’s palsy (ICD-10 code: G51.0) who began treatment within 72 h of symptom onset and had at least 6 months of follow-up data. Exclusion criteria included diagnosis of Ramsay Hunt syndrome (B02.21), central facial palsy (e.g., due to cerebrovascular disease), a history of previous Bell’s palsy, or receiving simultaneous care across multiple departments.

### 2.3. Treatment Group Classification

Patients were categorized into three groups based on the initial treatment department:

ENT Group: Received corticosteroid-based treatment, consisting of corticosteroids (e.g., prednisone) with or without antivirals (e.g., acyclovir). Typical protocols included prednisone (1 mg/kg/day, maximum 60 mg, for 5–10 days, tapered over 5 days) with or without acyclovir (400 mg five times daily for 7 days), though dosages varied by clinician [2]. Adjunctive treatments, such as analgesics (e.g., acetaminophen, ibuprofen) or eye lubricants, were prescribed as needed but were not consistently documented.

Pain Medicine Group: Received nerve blocks (e.g., stellate ganglion blocks), physical therapy, or neuromodulators (e.g., gabapentin). Treatments were tailored to pain or muscle dysfunction, with blocks administered 1–2 times weekly and physical therapy including electrical stimulation [15].

KM Group: Received acupuncture and herbal medicine. Manual acupuncture (30 min sessions, 2–3 times weekly) targeted facial nerve points, and herbal decoctions were customized based on patient symptoms [9].

### 2.4. Data Collection

Data included age, sex, comorbidities (hypertension, diabetes, hyperlipidemia, obesity, pregnancy, autoimmune disease, hypothyroidism), initial House–Brackmann (HB) grade, treatment modality, time to recovery, complications (synkinesis, dry eye, contracture), recurrence, and patient satisfaction (assessed via structured EMR entries). The HB grade, a validated scale for facial nerve function ranging from I (normal) to VI (total paralysis), was used to assess severity and recovery [16]. Synkinesis, involuntary muscle movements during voluntary actions, was evaluated clinically. Patient satisfaction was rated on a 5-point Likert scale in EMRs, though not validated [17].

### 2.5. Outcomes

The primary outcome was complete recovery, defined as achieving an HB grade of 1 within 6 months. Secondary outcomes included time to recovery (in weeks), recurrence, treatment-related complications, and patient satisfaction.

### 2.6. Propensity Score Matching and Quality Control

To reduce selection bias, 1:1:1 PSM was performed using logistic regression, with the treatment group (ENT, Pain Medicine, KM) as the outcome. Covariates included age, sex, comorbidities, and initial HB grade (IV vs. others). All eligible patients (*n* = 600) were pooled, and nearest-neighbor matching with a 0.2 caliper width created balanced triplets (200 per group). PSM minimized bias from patient self-selection of departments, as is common in South Korea. Sensitivity analyses with caliper widths of 0.1–0.3 confirmed robust matching [18]. Balance was confirmed by standardized mean differences (SMDs) < 0.1 and *p* > 0.05. Chi-square and Kruskal–Wallis tests verified post-matching balance (*p* > 0.05). Missing data (<5%) were handled via multiple imputation.

### 2.7. Statistical Analysis

Categorical variables were compared using chi-square or Fisher’s exact tests; continuous variables used Kruskal–Wallis tests with Bonferroni-adjusted Dunn’s post hoc tests. Effect sizes were calculated using Cohen’s d for continuous outcomes (e.g., time to recovery) and phi coefficients for categorical outcomes (e.g., complete recovery). Logistic regression assessed odds ratios (ORs) and 95% confidence intervals (CIs) for outcomes, adjusted for PSM covariates. Kaplan–Meier survival analysis with log-rank testing compared recovery times. Statistical analyses were performed using R (version 4.2.1) with packages ‘MatchIt’ for PSM, ‘survival’ for Kaplan–Meier analysis, and ‘stats’ for regression. Data preprocessing used Python (version 3.9) for cleaning and imputation [19]. A *p*-value < 0.05 was considered significant.

## 3. Results

### 3.1. Baseline Characteristics

Table 1 shows the baseline characteristics of 600 patients across the ENT, Pain Medicine, and KM groups after propensity score matching. The mean ages were 42.1 ± 12.4 (ENT), 46.7 ± 14.1 (Pain Medicine), and 44.5 ± 13.9 years (KM; *p* = 0.068). Covariates were balanced, confirming effective PSM (SMDs < 0.1). Patients aged ≥65 years accounted for 19.0%, 29.5%, and 22.5% (*p* = 0.081) of the respective groups, while females accounted for 54.0%, 61.0%, and 58.0% (*p* = 0.143). The rates of comorbidities, including hypertension, diabetes, hyperlipidemia, obesity, pregnancy, autoimmune disease, hypothyroidism, and initial HB Grade IV, showed no significant differences (*p* > 0.05).

### 3.2. Treatment Outcomes

Table 2 shows that the ENT group achieved the highest complete recovery rate (87.5%) compared to Pain Medicine (74.0%) and KM (69.5%) *(p* < 0.001; phi = 0.18, moderate effect). Median recovery time was shortest in the ENT group (4 weeks) versus the Pain Medicine (6 weeks) and KM groups (7 weeks) (*p* < 0.001; Cohen’s d = 0.65, ENT vs. KM, moderate to large effect). Pairwise comparisons confirmed that the ENT group outperformed the KM group in recovery time (*p* < 0.001), with smaller differences versus the Pain Medicine group (*p* = 0.045). Synkinesis was least frequent in the ENT group (6.0%) and was highest in the KM group (11.5%) (*p* = 0.027). Patient satisfaction was generally high across all groups but varied modestly (*p* = 0.049). Recurrence rates did not differ statistically among the groups (*p* = 0.856).

### 3.3. Predictors of Complete Recovery

Multivariate logistic regression analysis identified several significant predictors of complete recovery after propensity score matching (PSM). Patients treated in the ENT department had significantly higher odds of complete recovery (OR = 1.75, 95% CI: 1.29–2.37, *p* < 0.001), as did those receiving early corticosteroid treatment (OR = 1.95, 95% CI: 1.42–2.68, *p* < 0.001) and patients showing early signs of recovery (OR = 1.70, 95% CI: 1.26–2.29, *p* < 0.001). A lower initial House–Brackmann (HB) grade (I–III vs. IV) was also a significant predictor (OR = 1.58, 95% CI: 1.18–2.11, *p* = 0.002), as was the absence of comorbidities (OR = 1.40, 95% CI: 1.05–1.86, *p* = 0.022). Although early acupuncture showed a trend toward benefit (OR = 0.76, *p* = 0.076), it did not reach statistical significance. Age < 40 years and treatment in the Pain Medicine department were not significant predictors (Table 3).

### 3.4. Recurrence Risk Factors

In the multivariate model for recurrence risk factors after PSM, a history of recurrence (OR = 2.53, 95% CI: 1.71–3.75, *p* < 0.001) was the strongest predictor of recurrent events. Other significant factors included hyperlipidemia (OR = 8.20, 95% CI: 1.52–44.80, *p* = 0.025), hypertension (OR = 4.40, 95% CI: 1.62–11.95, *p* = 0.006), autoimmune disease (OR = 1.47, 95% CI: 1.05–2.05, *p* = 0.022), initial HB grade IV (OR = 1.71, 95% CI: 1.24–2.35, *p* < 0.001), and diabetes (OR = 1.40, 95% CI: 1.05–1.87, *p* = 0.021). Hypothyroidism also reached significance (OR = 1.37, *p* = 0.043). Age ≥ 65, sex, and obesity were not significantly associated with recurrence (Table 4).

### 3.5. Time to Complete Recovery by Department

**Figure 1:**
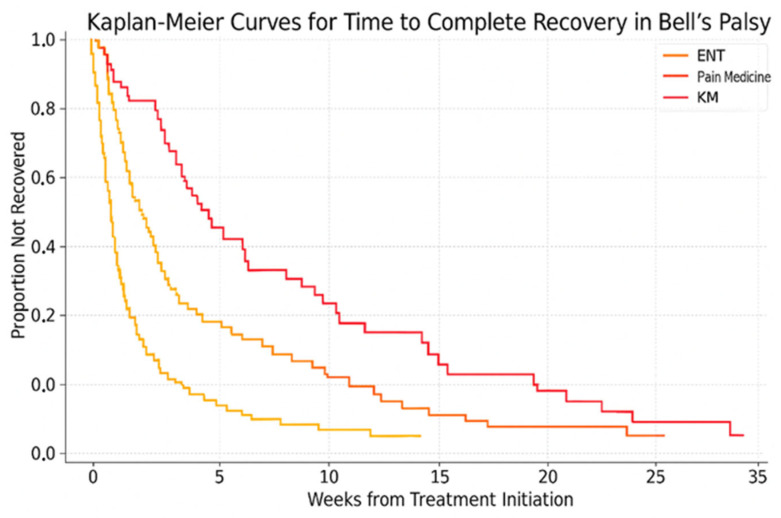
Kaplan–Meier curves for time to complete recovery in Bell’s palsy by department group. The ENT group demonstrated significantly faster recovery (median, 4 weeks) compared to the Pain Medicine group (6 weeks) and the KM group (7 weeks); log-rank *p* < 0.001. Abbreviations: ENT, Ear, Nose, and Throat; KM, Traditional Korean Medicine.

Figure 1 displays the Kaplan–Meier curves comparing time to complete recovery among the three treatment departments. The ENT group exhibited significantly faster recovery, with a median of 4 weeks, compared to the Pain Medicine (6 weeks) and KM groups (7 weeks). The difference among curves was statistically significant (log-rank *p* < 0.001).

### 3.6. Subgroup Analyses

Table 5 presents results from post hoc subgroup analyses. In patients with initial HB grade IV (*n* = 178), the ENT group had a 90% recovery rate (48/54) compared to 74% (49/66) in the Pain Medicine group and 65% (38/58) in the KM group (*p* < 0.01). For patients aged <40 years (*n* = 204), median recovery time was 3 weeks (interquartile range [IQR]: 2–5) in the ENT group (*n* = 68), 5 weeks (IQR: 3–7) in the Pain Medicine group (*n* = 70), and 6 weeks (IQR: 4–8) in the KM group (*n* = 66) (*p* < 0.05).

## 4. Discussion

This propensity-score-matched study demonstrates that corticosteroid-based treatment in the ENT department yields superior outcomes for Bell’s palsy, with an 87.5% complete recovery rate, a 4-week median recovery time, and a 6.0% synkinesis rate. These results, robust after PSM, were particularly pronounced in severe cases (HB grade IV, *n* = 178), where the ENT group achieved a 90% recovery rate compared to 74% in the Pain Medicine group and 65% in the KM group (*p* < 0.01). Similarly, younger patients (<40 years, *n* = 204) in the ENT group recovered faster (median, 3 weeks) than in the Pain Medicine (5 weeks) or KM group (6 weeks; *p* < 0.05), likely due to corticosteroids’ ability to reduce facial nerve inflammation in these subgroups [2,7,8]. The pronounced benefit in severe cases suggests that early corticosteroid-based treatment is particularly effective for patients with significant initial deficits, supporting targeted referral strategies [2,20].

The moderate effect size for complete recovery (phi = 0.18) indicates clinically meaningful differences, particularly for ENT treatment, which reduced recovery time by 2–3 weeks compared to KM. The Cohen’s d of 0.65 for recovery time (ENT vs. KM) suggests a substantial clinical advantage, likely improving quality of life by minimizing functional and psychosocial impacts [4]. These findings support early ENT referral, especially for severe cases and younger patients [15]. Patient satisfaction, though high across groups, was slightly lower in the Pain Medicine group, possibly due to slower recovery or invasive procedures like nerve blocks [17]. These findings align with clinical guidelines emphasizing corticosteroids as the cornerstone of Bell’s palsy management [2,7,8].

The superior outcomes in the ENT group support the hypothesis that viral reactivation (e.g., herpes simplex virus) and inflammation drive early pathology. Corticosteroids likely suppress edema, while antivirals may reduce viral replication, though dosage variations were not analyzed due to retrospective data limitations [1,6]. Future studies should explore dose–response relationships, as higher corticosteroid doses may enhance recovery in severe cases, while prolonged antiviral use could reduce recurrence [7].

Hypertension (OR: 4.40) and hyperlipidemia (OR: 8.20) were strong predictors of recurrence, suggesting that vascular risk factors increase facial nerve susceptibility, consistent with clinical evidence linking comorbidities to poorer outcomes [13,20]. These findings provide multivariate evidence in a matched cohort, highlighting modifiable targets for secondary prevention through metabolic control [15,20]. Hypothyroidism and autoimmune disorders also increased recurrence risk, indicating immune dysregulation as a contributor [4]. Clinicians should prioritize screening for and managing these comorbidities to reduce recurrence.

Compared to global studies, our findings align with Hohman et al. (2014), who reported corticosteroid benefits but did not compare departmental approaches [15]. Our study is novel in quantifying outcomes across ENT, Pain Medicine, and KM in South Korea’s pluralistic healthcare system, where patients choose specialties [11,14]. Integrated care models combining ENT’s pharmacological approach with KM’s acupuncture may optimize outcomes, particularly for patients preferring holistic treatments [9,11].

This study has several limitations inherent to its retrospective observational design. First, spontaneous recovery, reported in 70–85% of cases, is a significant confounder, as our treatment effects may partly reflect natural healing [3]. The absence of a no-treatment control group limits the isolation of treatment-specific effects. Patient choice of department may introduce selection bias, as KM patients may prefer non-pharmacological approaches [14]. Second, although PSM was used to reduce confounding, unmeasured or residual confounders, such as provider experience, patient preference, or socioeconomic status, may still have influenced the results [18]. Third, this study was conducted at a single tertiary care center in South Korea, which may limit its generalizability to other countries or healthcare systems with different care-seeking behaviors or treatment infrastructures Also, the single-center design, lack of validated satisfaction measures, and 6-month follow-up may miss delayed complications [1,4]. Variation in corticosteroid or antiviral dosages and unrecorded adjunctive treatments (e.g., analgesics) are additional limitations, as these may influence outcomes. Fourth, patient satisfaction data were collected through structured EMR fields, rather than through validated patient-reported outcome measures, which may have potentially underestimated the subjective experience [21]. Fifth, the 6-month follow-up period was insufficient to capture delayed complications or long-term psychosocial effects [1,4]. Sixth, treatment protocols within departments were not analyzed in detail, so variations in corticosteroid dosage, acupuncture frequency, or physical therapy methods could not be evaluated. Finally, although multiple imputation was used to handle missing data, this technique still introduces uncertainty, particularly in subgroup analyses [19].

Clinicians should prioritize early ENT referral for corticosteroid-based treatment within 72 h, particularly for severe cases, and screen for hypertension and hyperlipidemia to reduce recurrence risk. Integrated care models and metabolic interventions (e.g., lipid-lowering therapies) warrant further exploration [15,20].

## 5. Conclusions

Corticosteroid-based treatment in the ENT department significantly improves recovery outcomes for Bell’s palsy, particularly in severe cases, while comorbidities like hypertension and hyperlipidemia strongly predict recurrence. These findings underscore the need for early ENT referral, standardized treatment protocols, and proactive comorbidity management. Multicenter prospective trials with standardized dosages, longer follow-up, and integrated ENT–KM approaches are needed to validate these results and optimize global Bell’s palsy care.

## Figures and Tables

**Table 1:** Baseline characteristics by department (post-PSM, *n* = 600).

	Group	ENT (*n* = 200)	Pain Medicine (*n* = 200)	KM (*n* = 200)	*p*-Value
Variables					
Age, years		42.1 ± 12.4	46.7 ± 14.1	44.5 ± 13.9	0.068
Age ≥ 65 years		38 (19.0)	59 (29.5)	45 (22.5)	0.081
Female, *n* (%)		108 (54.0)	122 (61.0)	116 (58.0)	0.143
Hypertension		55 (27.5)	68 (34.0)	60 (30.0)	0.192
Diabetes		51 (25.5)	56 (28.0)	47 (23.5)	0.327
Hyperlipidemia		36 (18.0)	50 (25.0)	42 (21.0)	0.097
Obesity (BMI > 30)		38 (19.0)	45 (22.5)	40 (20.0)	0.317
Pregnancy, *n* (%)		6 (3.0)	4 (2.0)	7 (3.5)	0.725
Autoimmune disease		13 (6.5)	18 (9.0)	15 (7.5)	0.443
Hypothyroidism		19 (9.5)	23 (11.5)	21 (10.5)	0.690
Initial HB Grade IV		54 (27.0)	66 (33.0)	58 (29.0)	0.215

Notes: Data are expressed as the mean ± SD or *n* (%). *p*-values were calculated using chi-square tests for categorical variables and Kruskal–Wallis tests for continuous variables, comparing all three groups. Abbreviations: ENT, Ear, Nose, and Throat; KM, Traditional Korean Medicine; BMI, Body Mass Index; HB, House–Brackmann; PSM, propensity score matching; SD, standard deviation.

**Table 2:** Treatment Outcomes by Department (*n* = 600).

	Group	ENT (*n* = 200)	Pain Medicine (*n* = 200)	KM (*n* = 200)	*p*-Value	Effect Size
Variables						
Complete Recovery (HB 1, 6 mo)		175 (87.5)	148 (74.0)	139 (69.5)	<0.001	Phi = 0.18
Time to Recovery, weeks		4 [3–7]	6 [4–9]	7 [5–9]	<0.001	Cohen’s d = 0.65 (ENT vs. KM)
Synkinesis		12 (6.0)	17 (8.5)	23 (11.5)	0.027	Phi = 0.10
Patient Satisfaction		165 (82.5)	156 (78.0)	169 (84.5)	0.049	Phi = 0.08
Recurrence		7 (3.5)	9 (4.5)	8 (4.0)	0.856	Phi = 0.02

Notes: Data are expressed as the median [interquartile range] or *n* (%). *p*-values were derived from chi-square tests for categorical outcomes and Kruskal–Wallis tests for time to recovery, comparing all three groups. Bonferroni-adjusted Dunn’s tests were used for post hoc pairwise comparisons of time to recovery. Abbreviations: ENT, Ear, Nose, and Throat; KM, Traditional Korean Medicine; HB, House–Brackmann; mo, months; EMR, electronic medical record.

**Table 3:** Multivariate logistic regression analysis for the predictors of complete recovery (after PSM).

	OR	*p*-Value	95% CI
Age < 40 years	1.28	0.072	0.96–1.70
Early Acupuncture	0.76	0.076	0.57–1.02
ENT Department	1.75	<0.001	1.29–2.37
Pain Medicine Department	1.10	0.550	0.81–1.49
Early Corticosteroid Treatment	1.95	<0.001	1.42–2.68
Lower Initial HB Grade (I–III vs. IV)	1.58	0.002	1.18–2.11
Absence of Comorbidities	1.40	0.022	1.05–1.86
Early Signs of Recovery	1.70	<0.001	1.26–2.29

Notes: *p*-values and ORs were derived from logistic regression, adjusted for PSM covariates. Effect sizes (phi) were calculated for significant predictors. Abbreviations: PSM, propensity score matching; OR, odds ratio; CI, confidence interval; ENT, Ear, Nose, and Throat; HB, House–Brackmann.

**Table 4:** Multivariate logistic regression for recurrence risk factors (after PSM).

	OR	*p*-Value	95% CI
Age ≥ 65 years	1.12	0.992	0.87–1.45
Female (vs. Male)	1.75	0.345	0.83–3.70
Hypertension	4.40	0.006	1.62–11.95
Diabetes	1.40	0.021	1.05–1.87
Hyperlipidemia	8.20	0.025	1.52–44.80
Obesity (BMI > 30)	1.20	0.301	0.85–1.69
Hypothyroidism	1.37	0.043	1.00–1.88
Autoimmune Disease	1.47	0.022	1.05–2.05
Initial HB Grade IV (vs. I–III)	1.71	<0.001	1.24–2.35
History of Recurrence	2.53	<0.001	1.71–3.75

Notes: *p*-values and ORs were derived from logistic regression, adjusted for PSM covariates. Abbreviations: PSM, propensity score matching; OR, odds ratio; CI, confidence interval; BMI, Body Mass Index; HB, House–Brackmann.

**Table 5:** Subgroup analyses for recovery outcomes by department.

Subgroup	Variable	ENT	Pain Medicine	KM	*p*-Value
Initial HB Grade IV (*n* = 178)	Complete Recovery, *n* (%)	48/54 (90.0)	49/66 (74.0)	38/58 (65.0)	<0.01
Age < 40 years (*n* = 204)	Time to Recovery, weeks (IQR)	3 (2–5)	5 (3–7)	6 (4–8)	<0.05

Notes: Data are expressed as *n* (%) for recovery rates and median (IQR) for recovery times. *p*-values were derived from chi-square tests for recovery rates and Kruskal–Wallis tests for recovery times, comparing all three groups. Subgroup analyses were conducted post hoc on the PSM cohort. Abbreviations: ENT, Ear, Nose, and Throat; KM, Traditional Korean Medicine; HB, House–Brackmann; IQR, interquartile range; PSM, propensity score matching.

## Data Availability

The datasets analyzed are available from the corresponding author upon reasonable request.

## Abbreviations

**Abbreviation**	**Definition**
ENT	Ear, Nose, and Throat
KM	Traditional Korean Medicine
HB	House–Brackmann
PSM	Propensity Score Matching
OR	Odds Ratio
CI	Confidence Interval
EMR	Electronic Medical Record
ICD-10	International Classification of Diseases, 10th Revision
